# Trends and Hot Spots of Macrophages Linked to Metabolic Syndrome: A Comprehensive Bibliometric and Visualization Analysis (2014–2024)

**DOI:** 10.1155/mi/9487093

**Published:** 2025-05-24

**Authors:** Yuan Zhang, Qiwang He, Xiaoqian Cong, Huazhou Qiu, Chuan Hua

**Affiliations:** ^1^College of Traditional Chinese Medicine, Hubei University of Chinese Medicine, Wuhan 430061, Hubei, China; ^2^College of Acupuncture and Orthopedics, Hubei University of Chinese Medicine, Wuhan 430061, Hubei, China; ^3^Department of Mathematic Computing and Applied Mathematics Concentration, University at Buffalo, Buffalo 14260, New York, USA; ^4^Department of Endocrinology, Hubei Provincial Hospital of Traditional Chinese Medicine, Affiliated Hospital of Hubei University of Chinese Medicine, Wuhan 430061, Hubei, China; ^5^The First Clinical College, Hubei University of Chinese Medicine, Wuhan 430061, Hubei, China; ^6^Hubei Shizhen Laboratory, Wuhan 430061, Hubei, China

**Keywords:** bibliometric analysis, inflammation, macrophages, metabolic syndrome, visualization

## Abstract

**Background:** Metabolic syndrome (MetS) has been closely associated with macrophages, as evidenced by a substantial body of literature. However, a comprehensive bibliometric analysis of this research domain remains absent. This study aims to systematically assess the current state of research, identify emerging trends, and investigate key hot spots within macrophage-related MetS research from a bibliometric perspective.

**Methods:** Data including 1657 records on macrophages and their association with MetS were retrieved from the Web of Science Core Collection (WoSCC) database, covering the period from 2014 to 2024. The analysis was conducted using VOSviewer (v1.6.20), CiteSpace (v6.3.R1), the R package “bibliometrix” (v4.4.1), and Excel 2019.

**Results:** The annual number of publications peaked in 2017, 2018, and 2021, followed by a decline. However, the increasing citation count suggests growing recognition and influence of this research area. The United States and China account for over half of the academic output in this field, with strong collaborative networks positioning them as key contributors to its advancement. Yan Huang emerged as the most prolific author, while Gozal David had the highest co-citation frequency. Frontiers in Immunology was identified as the most active journal in this domain, whereas the Journal of Clinical Investigation recorded the highest citation impact. Keywords such as “inflammation,” “obesity,” and “insulin resistance” appeared most frequently, with “gut microbiota” showing the strongest citation bursts. Themes like inflammation, obesity, and expression need sustained attention and resource allocation, while themes such as macrophage activation syndrome, diagnosis, and mutations need a strategic reevaluation.

**Conclusion:** This study systematically evaluates the research landscape, priorities, and emerging trends of macrophages associated with MetS over the last decade, providing an overview of the field and valuable insights for researchers.

## 1. Introduction

Metabolic syndrome (MetS) has emerged as a global health concern, affecting an estimated over 1 billion individuals worldwide [1]. North America exhibits the highest prevalence of MetS at 27.93%, followed closely by South America at 27.65%. In contrast, Asia reports a slightly lower prevalence rate of 21.27%[2]. Among adults, the prevalence is notably high, with rates of 31.1% in the Chinese mainland and 37.1% in the United States [3, 4]. With advancements in societal development and rising living standards, the adoption of unhealthy dietary habits and sedentary lifestyles has contributed to an alarming yearly rise in the prevalence of MetS [5]. MetS is a critical risk factor for a range of chronic illnesses, including type 2 diabetes and cardiovascular and cerebrovascular diseases. Moreover, it is associated with other significant conditions, such as nonalcoholic fatty liver disease (NAFLD), reproductive disorders, and chronic kidney disease [6–9]. The health and socioeconomic burden posed by MetS and its related diseases highlights the urgent need to address this growing issue.

Over the past decade, significant progress has been made in comprehending the relationship between MetS and macrophages. Macrophages play an essential part in the development stage of MetS, particularly in the inflammatory processes linked to atherosclerosis, NAFLD, obesity, and other metabolic disorders. Emerging research indicates that the metabolic condition of macrophages influences their functionality and subsequently impacts the progression of MetS [10]. As a result, targeting the metabolic state of macrophages has gained attention as a potential therapeutic strategy. For example, supplementation with specific metabolites, such as eicosapentaenoic acid and docosahexaneoic acid, has been shown to restore macrophage function and promote tissue repair [11, 12]. In addition, advances in genetic engineering, such as the development of chimeric antigen receptors–macrophages, offer novel approaches to treating MetS [13, 14].

Despite these advancements, no comprehensive review has systematically analyzed the main areas of focus, current research trends, and thematic evolution in macrophage-related MetS studies. Bibliometric and visualization analyses have emerged as valuable research methodologies, using statistical and mathematical techniques to quantitatively evaluate the quality, volume, and citation impact of publications within a specific field [15–17]. These methods enable the extraction of comprehensive data on countries, institutions, authors, journals, references, and keywords, facilitating an understanding of academic collaboration networks, research hot spots, and emerging trends. This study aims to conduct an in-depth bibliometric and visualization analysis of macrophage-related MetS research from 2014 to 2024. By examining the current state of the field and identifying key trends and research priorities, this study intends to offer valuable insights into this critical area of investigation.

## 2. Methods

### 2.1. Source of Data and Search Methodology

Following the guidance of prior research, the Web of Science Core Collection (WoSCC) was selected as the primary database due to its reliability and comprehensive coverage for bibliometric analysis. The retrieval process was finalized on September 30, 2024, with the searches covering literature published between January 1, 2014 and September 30, 2024. All retrieved publications were sourced exclusively from WoSCC. The search strategy employed the following terms: TS = (Macrophage OR Histocyte OR Macrophages OR Histocytes) AND TS = (Syndrome, Metabolic X OR X Syndrome, Metabolic OR Metabolic Syndrome OR Syndrome, Metabolic OR Syndrome X, Metabolic OR Syndrome X, Reaven OR Metabolic Syndromes OR Insulin Resistance Syndrome X OR Metabolic Syndrome OR Syndromes, Metabolic OR Syndrome X, Insulin Resistance OR Reaven Syndrome X OR Cardiovascular Syndromes, Metabolic OR Metabolic Cardiovascular Syndrome OR Cardiovascular Syndrome, Metabolic OR Cardiometabolic Syndrome OR Syndromes, Cardiometabolic OR Syndrome, Metabolic Cardiovascular OR Cardiometabolic Syndromes OR Syndrome X, Dysmetabolic OR Dysmetabolic Syndrome X) AND Document types = (Article OR Review) AND Language = (English).

The literature selection process for this study was based on the following inclusion criteria: (1) Studies must directly address the association between macrophages and MetS. (2) Both human studies and animal or cell experiments are included. (3) The search results were limited to articles and reviews manuscript categories published in English. (4) Articles published between January 1, 2014 and September 30, 2024. The exclusion criteria included: (1) The topic was not related to macrophages and MetS. (2) Non-research materials such as editorial material, early access, book chapters, meeting abstract, proceedings paper, letters, and retracted publication. (3) Studies lacking scientific rigor, incomplete data, or flawed statistical analysis. (4) Duplicate publications. Two researchers, Yuan Zhang and Qiwang He, independently conducted the searches and screened the articles based on predefined inclusion and exclusion criteria. Cross-verification of the results was performed to ensure consistency, with Chuan Hua overseeing quality control and proofreading. Any disagreements were resolved through discussion until a consensus was reached.

**Figure 1:**
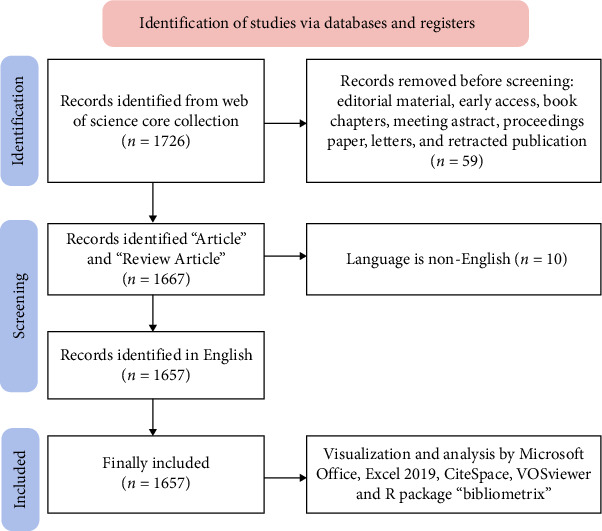
Flowchart of study selection.

Relevant data from the WoSCC database, including publication year, author names, national affiliations, institutional affiliations, journal titles, keywords, and references, were extracted and saved in download.txt files. These files were subsequently imported into Microsoft Excel 2019 on September 30, 2024, to ensure data accuracy and mitigate potential discrepancies. The study selection process is visually presented in Figure 1.

### 2.2. Data Analysis

We utilized VOSviewer (version 1.6.20), a widely recognized tool for extracting crucial information from large datasets of publications. VOSviewer is commonly used to construct networks related to collaboration, keyword co-occurrence and co-citation. In this study, its primary function was to analyze key entities such as countries/regions, institutions, authors, and keyword co-occurrence. The network diagrams generated by VOSviewer visually represent these entities as nodes. The node size indicates the frequency of occurrence, while node color signifies classification groups. Connections between nodes, represented as lines, indicate the degree of co-reference or collaboration, with line thickness reflecting the strength of these relationships. Additionally, we employed Citespace (version 6.3.R1), developed by Chen et al. [15], for further bibliometric analysis and visualization. CiteSpace was important in identifying the evolution of keywords over time, keywords with significant citation bursts, and constructing dual-map overlays to illustrate the evolutionary trajectory of the research domain within academic journals.

The R package “bibliometrix” (version 4.4.1) was used for the thematic area analysis of macrophages-related research linked to MetS.

Journal quartile rankings and impact factors (IFs) were obtained from the Journal Citation Reports (JCR) 2023 database to provide additional contextual insights. Additionally, the analysis of publication trends was facilitated by Microsoft Office Excel 2019 for quantitative insights.

## 3. Results

### 3.1. Analysis of Publication and Citation Trends

**Figure 2:**
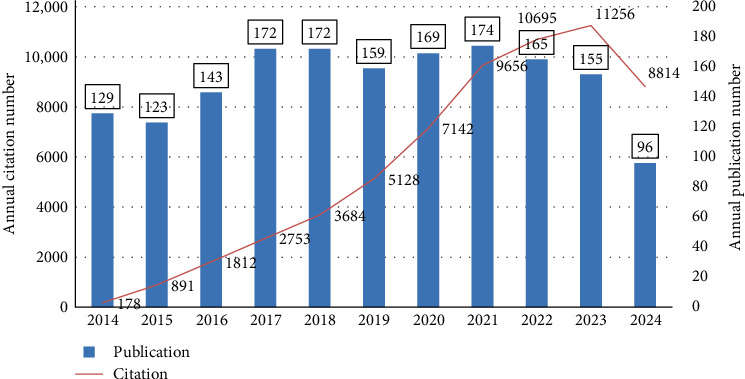
Yearly trends in publications and citations (2014–2024).

Through our systematic search, we identified a total of 1657 studies on macrophages associated with MetS covering the years 2014–2024. These included 1254 original research articles and 403 review papers.  Figure 2 illustrates the annual distribution of publications over this decade. Peak publication activity occurred in 2017, 2018, and 2021, while a noticeable decline in publication numbers is evident after 2021.

Despite these variations in article output, citation trends show a consistent upward trend over the years. Citations increased significantly, from only 178 in 2014 to an impressive 11,256 by the end of 2023. This pattern highlights the growing academic interest and influence of macrophage-related research in the context of MetS over the study period.

### 3.2. Analysis of Countries and Regions

The publications analyzed in this study originate from 71 countries and regions. As shown in Table 1, the top 10 contributors are distributed across North America, Asia, and Europe, with Europe (*n* = 6) and Asia (*n* = 3) being the most represented. Among these, the most productive country is the United States (*n* = 497, 30.66%), followed by China (*n* = 387, 23.87%), Japan (*n* = 151, 9.32%), and Germany (*n* = 109, 6.72%). Collectively, over half of the the total publications are from the United States and China.

**Figure 3:**
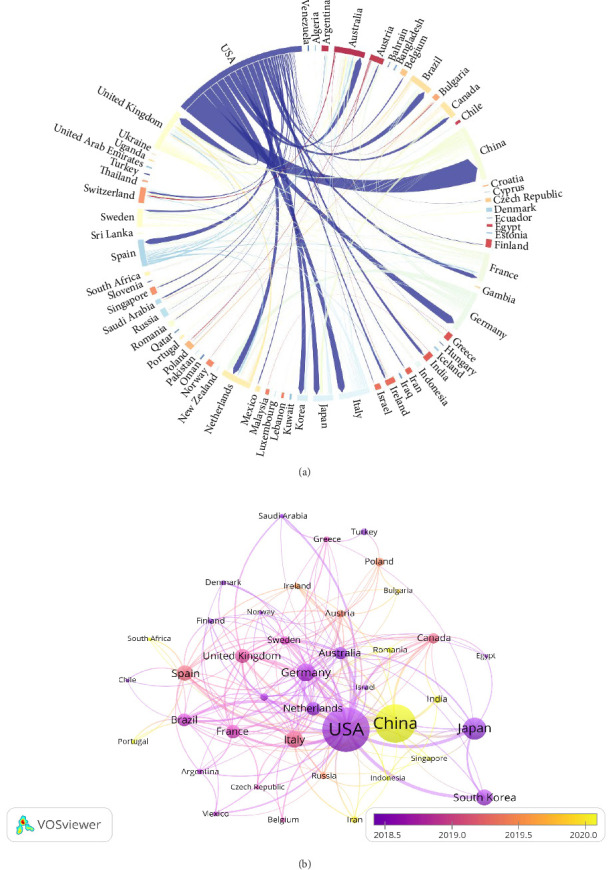
(A) Chord diagram illustrating international cooperation between countries and regions. The nodes are arranged along a circular path, with weighted lines connecting them. Each node represents a specific country or region and the length of the node's arc correlates with the quantity of publications from that country or region. The connecting lines indicate the extent of international collaboration; thicker lines signify stronger cooperative ties between nations. (B) Overlay network map of countries and regions. Node colors indicate the publication period, while lines indicate collaborative links. Each node represents a distinct country or region, with node size indicating to the number of publications.

Figure 3A illustrates a chord diagram depicting the collaborative relationships among countries and regions. Significant levels of international cooperation are evident. For instance, the United States demonstrates active collaborations with China, Japan, South Korea, Iran, and Germany. Similarly, China has established strong research partnerships with the United States, the United Kingdom, and Japan. Notably, China and the United States have the closest academic collaboration.

Figure 3B presents a temporal network map of countries and regions. A remarkable upward trend in academic publications from China is observed after 2020. While China currently ranks second to the United States in total publication volume, its consistent year-on-year growth underscores the increasing attention and activity in this research field within the country. In contrast, the United States, which historically laid the foundation for this field with a substantial number of early publications, has experienced a declining trend in recent years. This suggests that research priorities in the United States may be shifting toward other domains.

### 3.3. Analysis of Institutions

The top 11 institutions contributing to the research are distributed across four countries, with six located in the United States (Table 2). Among these, Harvard Medical School stands out with the most relevant publications (*n* = 30), closely followed by Shanghai Jiao Tong University (*n* = 28) and the University of São Paulo (*n* = 27). Notably, the University of California, San Diego, has the highest citation frequency, highlighting the impact of its published work.

**Figure 4:**
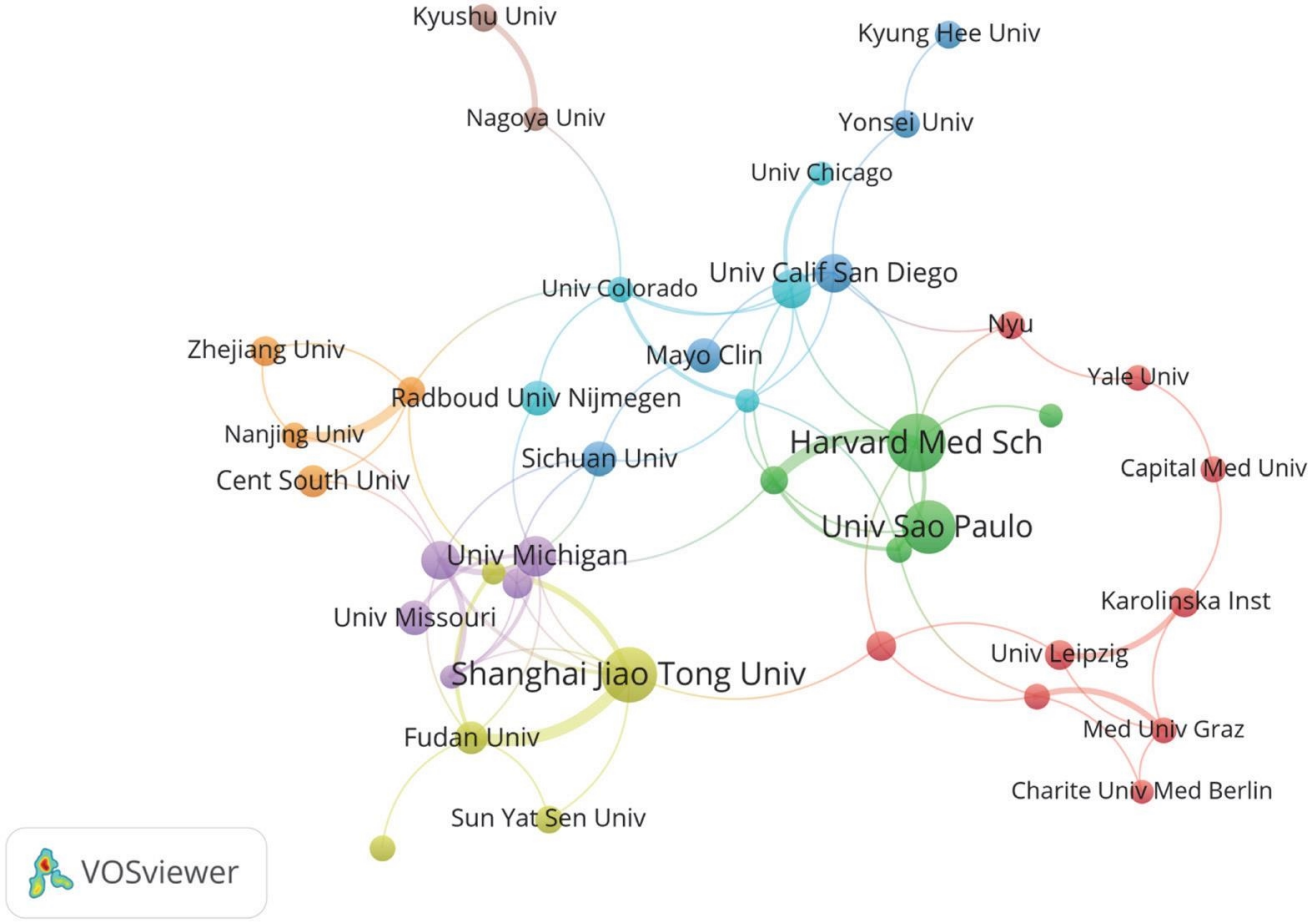
Collaboration network map of the institutions from 2014 to 2024. The nodes represent individual institutions, with the node size indicating the volume of publications. Colors denote the clusters to which institution belong, while the connecting lines illustrate collaborative relationships.

Analysis using VOSviewer reveals distinct yet interconnected clusters that represent institutional collaborations (Figure 4). A particularly strong collaborative network exists between the University of California, San Diego, and Harvard Medical School. Similarly, Shanghai Jiao Tong University, Sun Yat-sen University, and Fudan University demonstrate a strong alliance. Furthermore, institutions such as Karolinska Institute, Medical University of Graz, and Charité-Universitätsmedizin Berlin demonstrate well-established and cohesive partnerships, emphasizing their significant contributions to the field.

### 3.4. Analysis of Authors

**Figure 5:**
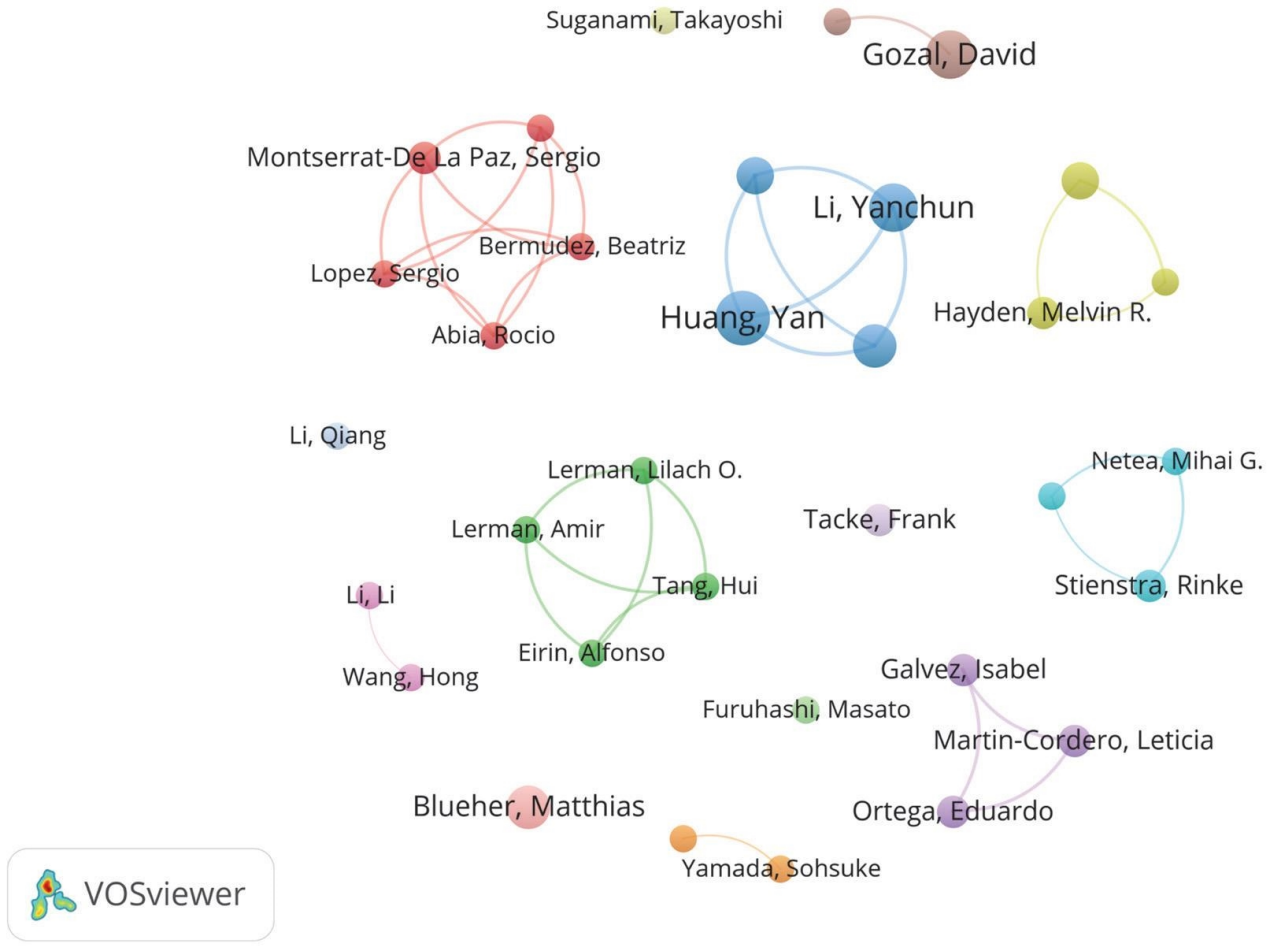
The authors' collaboration network map.

The analysis reveals that Yan Huang has produced the most publications, while David Gozal is the most frequently cited author. Notably, three scholars including David Gozal, Matthias Blueher, and James R. Sowers, rank among the top 10 for both publication volume and citation frequency, as detailed in Table 3. Collaboration among authors has resulted in the formation of several distinct research groups (Figure 5). The nodes represent individual authors, while lines denote their collaborative relationships. These collaborative networks tend to be stronger among researchers who are geographically close or share similar cultural backgrounds, highlighting the influence of regional and cultural factors on academic partnerships.

### 3.5. Analysis of Journals

A total of 1657 papers were published across 639 journals, with 79 journals featuring five or more publications. Among the top 10 journals by publication volume, Frontiers in Immunology (IF = 5.7, *Q*1) led with 67 articles (*n* = 67), followed by the International Journal of Molecular Sciences (IF = 4.9, *Q*1; *n* = 59), and PLoS One (IF = 2.9, *Q*1; *n* = 43). Notably, Frontiers in Immunology emerged as the most frequently co-cited journal.

Among the journals analyzed, three journals have an IF above 5, with Cell (IF = 45.5, *Q*1) having the highest IF. Additionally, nine journals are rated within the *Q*1 category of the JCR. Details are provided in Table 4.

Regarding co-citation analysis, Journal of Clinical Investigation has the most co-citations (2883), followed by Diabetes (2669) and Nature (2367). These findings are summarized in Table 5.

**Figure 6:**
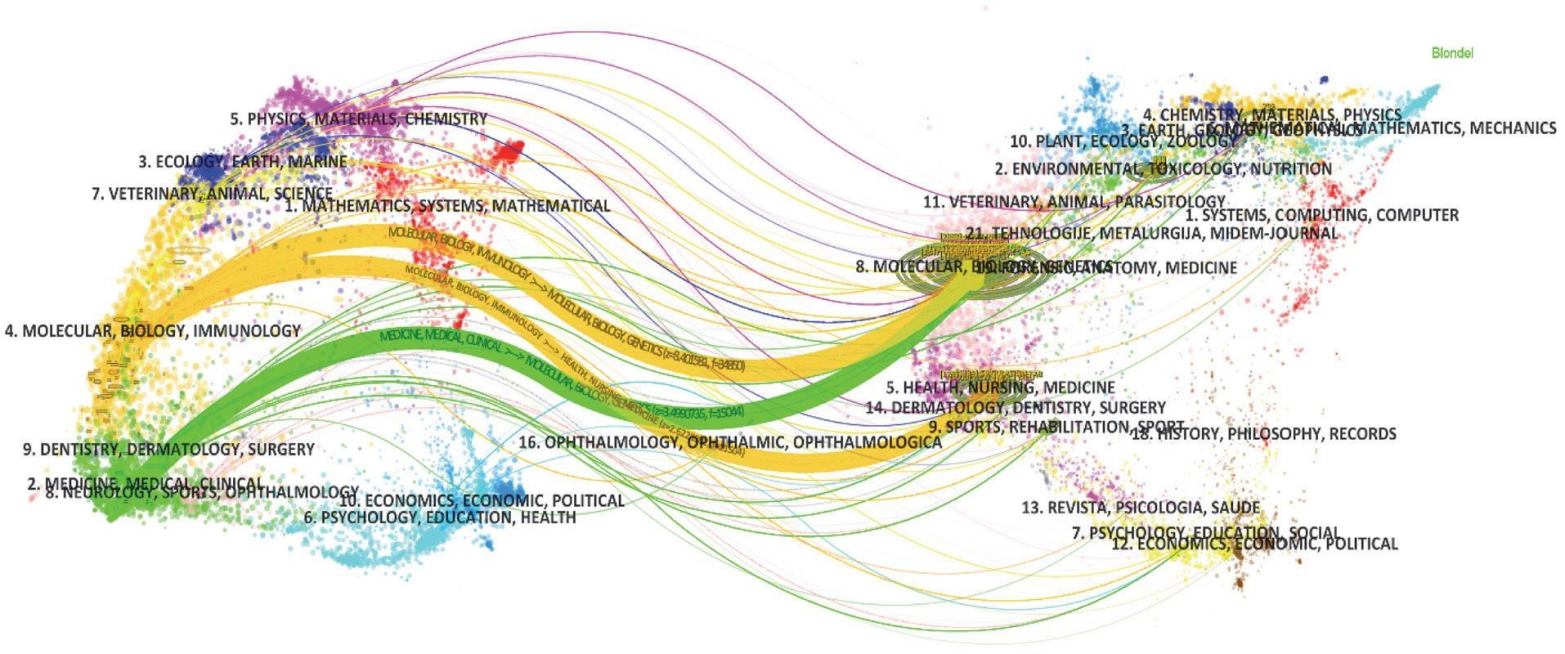
The dual-map overlay showcases the correlation between journals focusing on macrophages associated with MetS. The left panel represents the clustering of journals that cite relevant research, while the right panel displays the clustering of journals that are cited. The central curve represents the citation trend, where the vertical axis of an ellipse indicates the publication volume of a journal and the horizontal axis reflects the number of contributing authors. A larger vertical axis suggests a higher publication count, whereas a longer horizontal axis denotes broader author participation.

The double graph overlay analysis, which visualizes the relationships between citing and cited journals, indicated three main citation pathways (Figure 6). The first pathway shows that citing journals in the areas of molecular, biology, and immunology, predominantly cite journals in health, nursing, and medicine, as well as molecular, biology, and genetics. The second pathway highlights that citing journals in medicine, medical, and clinical are mainly derived from the fields of molecular, biology, and genetics.

### 3.6. Analysis of Highly Cited References

The top 15 co-cited articles listed in Table 6 reflect pivotal contributions to understanding the interplay between immune dysregulation, metabolic dysfunction, and therapeutic interventions. The most cited reference is “Inflammation as a link between obesity, MetS, and type 2 diabetes” (cited 1331 times), followed by “Vitamin C and Immune Function” (cited 973 times), and “Liver inflammation and fibrosis” (cited 815 times). In addition, the “Main Essence” section in Table 6 provides a summary of the core content of each highly cited reference.

### 3.7. Analysis of Keyword Co-Occurrence

Keywords provide critical insights into the emerging hot topics and research frontiers associated with macrophages in the context of MetS.  Table 7 highlights 13 keywords with an occurrence frequency of ≥100. Beyond foundational terms such as “metabolic syndrome” and “macrophage,” the most frequently occurring keywords are “inflammation” (634 occurrences), “insulin-resistance” (551 occurrences), and “obesity” (533 occurrences).

**Figure 7:**
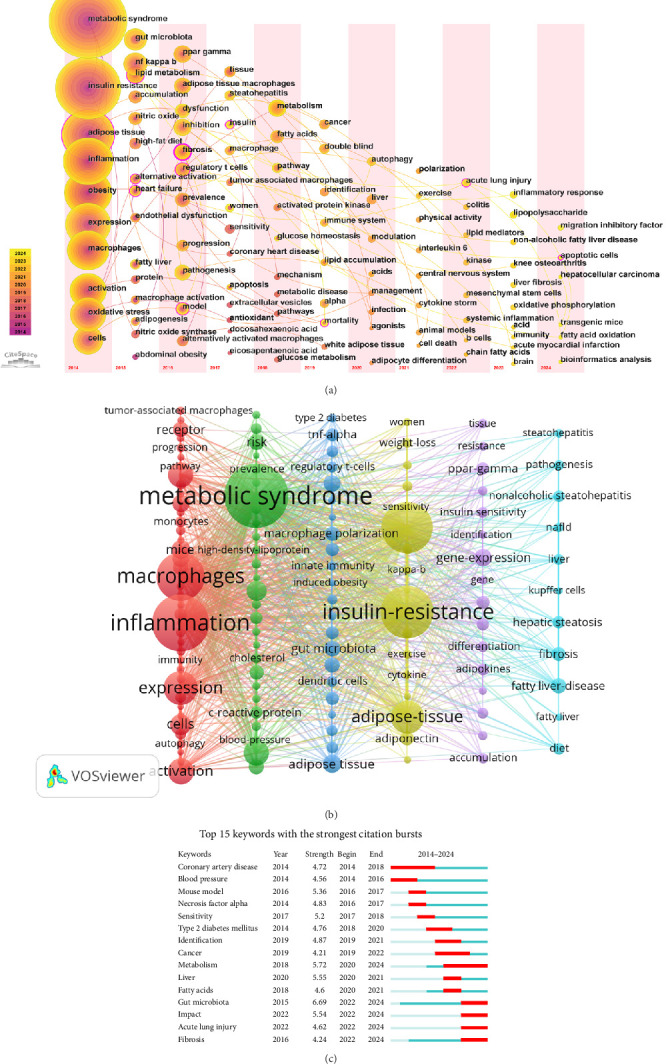
(A) A keyword timezone map. The keywords are arranged in different time zones according to the timeline, showing the evolution of keywords over time. Researchers can clearly observe the frequency and trend of keyword occurrence in different time periods. Through nodes (representing keywords) and lines (representing co-occurrence relationships), the timeline diagram intuitively displays the associations and mutual influences among keywords. (B) A collaborative keyword network visualization. Keywords are represented as nodes. The size of each node shows how frequently it appears, with larger nodes denoting more frequent keywords. The connections between nodes signify co-occurrence relationships and the thickness of the connecting lines indicates the strength of these associations. (C) Top 15 keywords with the strongest citation bursts.

Figure 7A presents a keyword timezone map, clearly illustrates the dynamic evolution of research priorities from 2014 to 2024. In the early research phase (2014–2016), the focus was primarily on the fundamental role of macrophages in MetS and their association with inflammation, with key terms including “metabolic syndrome,” “macrophages,” “insulin resistance,” “inflammation,” “adipose tissue,” and “obesity.” Subsequently, research gradually shifted towards more specific pathological mechanisms (2017–2020), especially liver-related complications such as “fatty liver” and “fibrosis.” Meanwhile, attention to macrophages regulation mechanisms and potential therapeutic targets also increased, with related terms including “apoptosis,” “autophagy,” and “polarization.” In recent studies (2021–2024), the focus has further deepened, mainly shifted to systemic inflammation and its extensive impacts, such as “acute lung injury,” “colitis,” “inflammatory response,” “lipopolysaccharide,” “interleukin-6,” and “cytokine storm.” In addition, lifestyle interventions, computational methods, and genetic models have gradually become hot spots, with related terms including “exercise,” “physical activity,” “bioinformatics analysis,” and “transgenic mice.” This trend indicates that research on macrophages linked to MetS has gradually shifted from the early exploration of basic mechanisms to clinical applications and multidisciplinary interdisciplinary research.

To further explore the relationships between keywords, 133 keywords with at least 20 occurrences were subjected to cluster analysis using VOSviewer. This analysis identified six distinct clusters, each representing a specific research domain, as depicted in Figure 7B. The red cluster focuses on underlying mechanisms, including terms such as “inflammation,” “oxidative stress,” and “autophagy.” The green cluster centers on MetS, with terms like “atherosclerosis” and “diabetes.” The yellow cluster highlights physiological or pathological phenomena, including terms such as “insulin resistance,” “obesity,” and “adipose tissue.” The dark blue cluster represents immunological and microbiological aspects, featuring terms such as “T-cells” and “gut microbiota.” The purple cluster is associated with gene expression and regulation, with keywords like “gene-expression” and “PPAR-γ.” Last, the light blue cluster focuses on liver health and disease, including terms such as “liver,” “nonalcoholic steatohepatitis,” and “fibrosis.” These clusters not only highlight specific research areas but also reveal their complex interrelationships. For example, there is a significant association between “inflammation” and “gut microbiota.” Inflammation is one of the core features of MetS, while gut microbiota regulate the activation and polarization of macrophages through their metabolites, such as short-chain fatty acids, thereby influencing systemic inflammatory responses [33]. For instance, short-chain fatty acids like butyrate can promote an anti-inflammatory macrophage phenotype, thereby alleviating inflammatory responses [34]. In contrast, metabolites like trimethylamine N-oxide promote a pro-inflammatory macrophage phenotype, exacerbating inflammatory responses [35]. This interaction underscores the importance of understanding how microbial metabolites regulate systemic inflammation and metabolic homeostasis. Similarly, the relationship between “insulin resistance” and “adipose tissue macrophages (ATMs)” has been extensively studied, with evidence indicating that macrophage-derived exosomes play a key role in regulating insulin sensitivity [36]. These interactions highlight the integrality of macrophage-related MetS research and suggest that future studies should concentrate on the relationship between metabolic, inflammatory, and microbial pathways.

Figure 7C presents the top 15 keywords with the strongest citation bursts. For instance, in 2014, keywords such as “coronary artery disease” and “blood pressure” experienced high citation density, reflecting intense focus on cardiovascular diseases during that period. Meanwhile, the keyword “gut microbiota” exhibited the highest emergence strength, with a rapid rise in research activity between 2022 and 2024. Similarly, the keyword “metabolism” ranked second, with sustained attention and growth from 2020 to 2024, reflecting its relevance and expanding influence in the field.

### 3.8. Analysis of Strategic Prioritization of Research Themes

**Figure 8:**
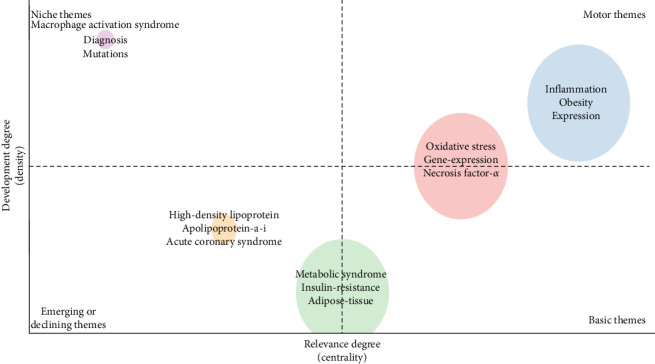
Thematic map illustrating the distribution of Keyword Plus in publications from 2014 to 2024. The horizontal axis represents centrality, while the vertical axis indicates density. In the top-right quadrant, motor themes emerge as both highly significant and extensively explored. In contrast, the top-left quadrant included well-developed but isolated themes, which, despite their current importance, appear disconnected from the broader research landscape. Shifting to the bottom-left quadrant, we identify emerging or declining themes including marginal topics with limited development potential that may either gain prominence or fade away. Finally, the bottom-right quadrant features basic yet transversal themes, which, although integral to the field, remain under explored.

Figure 8 illustrates the thematic map derived from Keyword Plus, constructed via the Conceptual Structure feature within the R package “bibliometrix”. The blue cluster, encompassing themes like inflammation, obesity, and expression, is characterized by its significance and maturity, thereby positioning these topics as salient and dynamic research domains. Sustained attention and resource allocation are imperative to further elucidate and expand the applications of these subjects. The red cluster, which incorporates themes such as oxidative stress, gene expression, and necrosis-factor-alpha, although fundamental, signifies areas necessitating additional scrutiny and refinement for a more holistic comprehension. The orange cluster, featuring emerging themes like high-density-lipoprotein, apolipoprotein-a-i, and acute coronary syndrome, along with the green cluster, containing MetS, insulin resistance, and adipose tissue, points to prospective avenues for future research endeavors. Vigilant surveillance of these clusters may yield crucial insights into emergent trends and innovations. Ultimately, the purple cluster, comprising themes such as macrophage activation syndrome, diagnosis, and mutations, underscores topics that merit reappraisal to ascertain their pertinence within the realm of macrophages associated with MetS. This cluster potentially encompasses areas where current knowledge is either stagnant or of diminished significance, thus, necessitating a strategic reevaluation.

## 4. Discussion

### 4.1. General Analysis

This study is based on an analysis of 1657 articles authored by 11,559 authors from 6735 institutions, retrieved from the WoSCC database for the period from January 1, 2014 to September 30, 2024.

The volume of publications peaked in 2017, 2018, and 2021, but has shown a declining trend thereafter. This pattern may suggest that as research on macrophages and MetS has advanced, foundational questions may have been addressed, leaving more complex and challenging issues to resolve. Additionally, external factors such as disruptions caused by the COVID-19 pandemic may have significantly impacted research activities, particularly in fields requiring extensive laboratory work and clinical data collection [37, 38]. Many research institutions faced temporary closures, reduced laboratory capacity, and reallocation of resources to COVID-19-related studies, which likely contributed to the decline in publications observed after 2021 [39, 40]. Despite these challenges, a substantial increase in citation counts during this period indicates the growing influence and sustained relevance of research in this field. This suggests that while the pandemic may have temporarily slowed the pace of new publications, the scientific community continues to engage with and build upon existing research.

In analyzing contributions by countries and regions, North America, Asia, and Europe emerged as the major contributors of scientific output. The United States remains the leading contributor, followed closely by China, with significant input also coming from Japan and Germany. Close scientific cooperation between nations is notable, particularly the robust collaboration between the United States and China.

Among the top 11 institutions ranked by publication output, more than half are in the United States. This may be attributed to the high prevalence of MetS in the country, which drives laboratory and clinical research on related topics. However, the linear characteristics of the interinstitutional collaboration network suggests that partnerships among institutions remain limited in diversity, highlighting the need for more extensive and multidisciplinary collaborations.

The most published author is Professor Yan Huang, who has extensively researched GPR40, a G protein-coupled receptor for free fatty acids [18]. Professor Yan Huang's team demonstrated that GPR40 activation in macrophages exerts anti-inflammatory and anti-osteoclastogenic effects. They were the first to show that GPR40 may play a therapeutic role in LDL receptor-deficient mice with hyperlipidemia-associated nonalcoholic steatohepatitis [19]. Furthermore, their research revealed that GPR40 positively influences MetS-associated periodontitis [20]. Meanwhile, Professor David Gozal is the most cited author. He discovered that PPAR*γ* and p62/SQSTM1 as key proteins that support lipid metabolism and reduce inflammation in metabolically active macrophages [32]. This article boasts an IF of 27.7 and is highly cited.

In the context of macrophages and MetS, the journal Frontiers in Immunology has the highest number of publications, highlighting its reputation and popularity in this research area. Among the top 10 journals, Cell has the highest IF, while the Journal of Clinical Investigation has the highest citation count. Notably, Nature has the highest IF among journals with the most citations. Both PLoS One and Cell are among the top 10 for both publication output and citation frequency, reflecting their dual roles in foundational and cutting-edge research. Foundational fields such as genetics and molecular biology provide the theoretical basis for medical and clinical research, which continues to innovate and apply this knowledge. Conversely, the demands of medical and clinical treatments drive advancements in foundational scientific fields.

Highly cited references represent foundational studies that have significantly shaped the research landscape of macrophages linked to MetS. The study by Esser et al. [18], published in 2014, is the most cited article. It revealed how macrophage-driven chronic inflammation in adipose tissue exacerbates insulin resistance and systemic metabolic dysregulation, laying the foundation for subsequent explorations of the complex mechanisms [18]. Ranking second is the study published in Nutrients by Carr and Maggini [19], which revealed the role of micronutrients in modulating macrophage activity. This work closely linked nutritional science with immunology, providing inspiration for clinical trials targeting macrophage metabolism through dietary interventions. The third-ranked study “liver inflammation and fibrosis” identified distinct macrophage subsets driving fibrosis and proposed therapeutic strategies to inhibit profibrotic signaling. Its emphasis on liver-specific macrophage polarization has influenced the direction of subsequent research on organ-specific metabolic inflammation [20]. In addition, a paragon of translational research was published by Marchetti et al. [32]. By demonstrating that NLRP3 inflammasome inhibition improves insulin sensitivity, this study highlighted the potential of modulating inflammasome activity in the treatment of MetS [32]. Notably, the study by Shen et al. [21], published in 2020 on the “Proteomic and Metabolomic Characterization of COVID-19 Patient Sera” revealed systemic metabolic reprogramming during viral infection, including alterations in macrophage lipid metabolism and cytokine profiles. Although not directly focused on MetS, this study provided mechanistic insights into how acute inflammation exacerbates preexisting metabolic disorders, further emphasizing the bidirectional relationship between immune activation and metabolic dysfunction [21].

Looking at the thematic evolution of highly cited references, early studies primarily focused on basic mechanisms. For instance, the three most cited articles published in 2014 all centered on the intrinsic links between inflammation and metabolic dysfunction. In contrast, recent studies have gradually shifted towards more complex microenvironmental factors. This trend is exemplified by research on the “Gut microbiota–bile acid–interleukin-22 axis orchestrates polycystic ovary syndrome” which explored the potential role of the gut microbiota in regulating macrophage polarization, consistent with the trend shown in Figure 7C [28]. Overall, these references highlight three key themes: inflammation as a central mediator of metabolic dysfunction; the significant role of microenvironmental factors (such as diet and microbiota) in shaping macrophage behavior and the translation of mechanistic insights into targeted therapeutic strategies. These studies have not only advanced basic scientific understanding but also provided new directions for clinical applications.

### 4.2. Frontiers and Hot Spots

Keywords enable us to promptly graspe the distribution and evolution of hot spots of macrophages linked to MetS. As shown in Table 7, keywords with an occurrence frequency of ≥100, such as “inflammation,” “insulin-resistance,” and “obesity,” highlight popular themes within this research domain. Temporal trends and citation bursts reveal nuanced shifts in research priorities, reflecting both methodological advancements and evolving mechanistic insights. The keywords timezone in Figure 7A not only reflects the changing interests of researchers in different mechanisms and factors, but also illustrates the continuous evolution of research methods. This transformation highlights the increasing complexity of the field, as well as the importance of translating basic science into clinical and lifestyle intervention measures. Figure 7B further categorizes the research landscape into six clusters, representing distinct thematic trends: mechanisms, MetS, physiological and pathological phenomena, immunological and microbiological aspects, gene expression and regulation, and liver health and disease. Collectively, these findings not only reflect current focal points in macrophage-related MetS research but also indicate potential directions for future studies, as detailed below.

### 4.3. Inflammation-Driven Obesity and Insulin Resistance: Macrophage-Mediated Mechanisms in MetS Pathogenesis

It is well-documented that obesity-induced chronic and low-grade inflammation contributes to insulin resistance, with macrophages are instrumental in this process [18, 41]. By secreting inflammatory molecules such as IL-6, IL-1*β*, and TNF-*α*, macrophages drive inflammation, exacerbating obesity, insulin resistance, and type 2 diabetes [42–44]. Moreover, metabolic byproducts regulate macrophage activation states, indicating a close link between metabolic and inflammatory pathways [45–47].

In obesity, elevated nutrient levels result in adipocyte hypertrophy and cell death [48, 49], which perpetuate chronic inflammation in adipose tissue. This inflammatory milieu induces a shift in macrophage polarization from the anti-inflammatory M2 phenotype to the pro-inflammatory M1 phenotype [50, 51]. The resulting inflammatory environment subsequently impacts other metabolic organs, including the liver and skeletal muscle, exacerbating insulin resistance by impairing glucose uptake in muscle and increasing hepatic glucose output. Dietary factors and fatty acids released from adipocytes further modulate the metabolic profile of ATMs, which accumulate in white adipose tissue during obesity. ATMs not only clear lipids released by necrotic adipocytes [42, 52] but also secret exosomes containing miRNA, influencing insulin sensitivity both in vitro and in vivo [53, 54]. The development and activation of macrophages in adipose tissue are therefore central to the pathogenesis of obesity-related metabolic diseases, emphasizing their close association with inflammation [55].

### 4.4. Gut Microbiota: A Rising Star in Mechanistic and Translational Research

Figure 7C identifies “gut microbiota” exhibited the strongest citation burst (2022–2024), driven by technological breakthroughs, mechanistic discoveries, and clinical relevance [56]. Advances in multi-omics approaches (e.g., metagenomics and metabolomics) have enabled comprehensive profiling of microbial communities and their interactions with host immunity [57]. Specific taxa (e.g., actinobacteria and bacteroidetes) and metabolites (e.g., trimethylamine N-oxide and short-chain fatty acids) were identified as key regulators of macrophage polarization and systemic inflammation [33]. Mechanistic studies highlight bidirectional crosstalk: microbial metabolites modulate macrophage metabolism via receptors like GPR43 and GPR109A, while macrophages shape microbiota composition through antimicrobial peptide secretion [34, 58]. Dysbiosis-driven macrophage activation exacerbates insulin resistance and hepatic steatosis, positioning gut microbiota as a therapeutic target [59]. Clinical trials further validate this potential—probiotics, prebiotics, and fecal transplantation improve metabolic parameters in MetS patients [60, 61]. The COVID-19 pandemic accelerated interest in this area, as systemic inflammation and immune–metabolic cross talk gained prominence [21].

### 4.5. Metabolic Reprogramming: Challenges and Opportunities

Metabolic reprogramming is a cell's “survival strategy” when facing stress. It alters energy metabolism to adapt to harsh environments. This is a hot topic in current biomedical research and plays a key role in various pathological states like cancer and immune diseases. In basic research, metabolic reprogramming has yielded fruitful results. For example, it has revealed the “Warburg effect” in cancer cells and the metabolic changes in immune cells when activated, offering a new perspective on disease mechanisms [62–64]. However, despite showing great therapeutic potential in animal experiments, it faces numerous challenges in clinical translation [65]. Metabolic reprogramming is a highly complex and dynamic network, its heterogeneity and plasticity make single-target drugs ineffective and the large metabolic differences among patients further complicate drug development [66, 67]. In clinical trials, insufficient drug specificity and metabolic distribution differences often lead to poor efficacy or severe side effects [68]. Moreover, targeting metabolic reprogramming may disrupt normal cell metabolism, causing unpredictable side effects and making regulatory approval very strict [69]. However, we should recognize the hope for the clinical translation of metabolic reprogramming. Through multidisciplinary collaborative innovation, in-depth understanding of its heterogeneity and dynamics, precise identification of key targets, and optimized clinical trial design, we can advance metabolic reprogramming from basic research to clinical application, opening up new treatment avenues for various diseases.

### 4.6. Metabolic Memory: A Novel Dimension in Macrophage-Driven MetS

Emerging evidence highlights “metabolic memory” as a critical mechanism linking sustained metabolic disturbances to long-term complications in MetS and diabetes [70]. Even after blood sugar is controlled, the metabolic memory of hyperglycemia can continue to affect the development of complications through epigenetic mechanisms. This phenomenon refers to the persistence of cellular dysfunction even after normalization of metabolic parameters, driven by epigenetic modifications, mitochondrial dysfunction, and chronic low-grade inflammation [71]. Macrophages, with their plasticity and role in maintaining inflammatory homeostasis, are central to this process [72]. For instance, prolonged hyperglycemia or lipid overload induces epigenetic alterations in macrophages, such as DNA methylation and histone modifications, which perpetuate pro-inflammatory phenotypes (e.g., sustained NF-*κ*B activation) and impair insulin signaling pathways [73, 74]. Integrating metabolic memory into macrophage biology offers novel insights into the chronicity of MetS and potential strategies to reset immune–metabolic homeostasis.

### 4.7. Multi-Omics Integration: Unraveling Macrophage Heterogeneity in MetS

The application of multi-omics approaches (transcriptomics, proteomics, and metabolomics) has revolutionized our understanding of macrophage diversity and function in MetS. Single-cell RNA sequencing has identified distinct macrophage subpopulations in adipose tissue, such as lipid-associated macrophages and metabolically activated macrophages, each with unique transcriptional profiles and metabolic dependencies [75, 76]. Proteomic analyses reveal that macrophages in obese adipose tissue exhibit upregulated glycolytic enzymes and downregulated oxidative phosphorylation proteins, aligning with their pro-inflammatory polarization [77]. Metabolomic studies further link gut microbiota-derived metabolites (e.g., succinate and itaconate) to macrophage metabolic reprogramming via succinate receptor 1 (SUCNR1) and itaconate-dependent Nrf2 activation [78, 79]. These multi-omics datasets not only identify biomarkers, for example, circulating miR-155 from macrophage exosomes, but also uncover therapeutic targets, such as mTORC1 inhibitors to normalize macrophage glycolysis [80, 81]. Future research should leverage spatial omics to map macrophage–microenvironment interactions in MetS-associated tissues, bridging molecular insights with clinical outcomes.

### 4.8. Bioinformatics Analysis: Unveiling Molecular Networks and Predictive Insights

Building upon the advancements in multi-omics integration, bioinformatics analysis has emerged as a pivotal tool for deciphering the molecular complexity of macrophage-associated MetS. It does more than just interpret data, playing a key role in the discovery and validation of therapeutic targets within the intricate molecular networks of MetS. By leveraging computational algorithms, machine learning, and network modeling, researchers are now able to systematically analyze high-dimensional datasets to identify key regulatory networks and predictive biomarkers. Recent studies have employed weighted gene co-expression network analysis to uncover hub genes like IL-6, VEGFA, STAT3, and PTGS2 that orchestrate macrophage polarization in obese adipose tissue [82, 83]. Similarly, pathway enrichment analyses have highlighted the centrality of the immune system in MetS and provided mechanistic insights into therapeutic targeting. For instance, BTK inhibitor ibrutinib may serve as a novel therapeutic agent for MetS, which shows effectiveness in reducing inflammation caused by macrophage accumulation [84]. The application of machine learning algorithms, such as random forest and deep neural networks, has further enhanced predictive capabilities. These tools enable the identification of macrophage-specific gene signatures associated with insulin resistance or hepatic steatosis, offering potential diagnostic markers [85, 86]. Despite these advancements, challenges remain in translating bioinformatics findings into clinical practice. Issues such as dataset heterogeneity, algorithm interpretability, and validation in diverse cohorts necessitate collaborative efforts between computational biologists and experimental researchers. Future directions may focus on developing user-friendly platforms for real-time data integration and leveraging artificial intelligence to predict therapeutic responses. As bioinformatics continues to bridge mechanistic discovery and translational applications, it holds immense potential to redefine our understanding of macrophage heterogeneity in MetS and accelerate the development of targeted therapies.

### 4.9. Trends in Future Research

The analysis presented above highlights the critical need to forecast future directions on macrophages associated with MetS. Future research may focus on how precise regulatory strategies targeting the metabolic states of macrophages and their clinical applications. This includes exploring the subtle mechanisms of macrophage activation, the influence of gut microbiota on macrophage functionality and the potential for metabolic reprogramming to modulate inflammatory responses. These efforts are crucial for advancing our understanding of MetS and developing more effective therapeutic interventions.

Looking ahead, future research trends are expected to converge at the intersection of key scientific clusters. Investigating the underlying mechanisms governing MetS, including its physiological and pathological processes, immune responses, microbiological interactions, gene expression, and liver health, holds immense potential. Integrating insights from these diverse fields could facilitate the development of more holistic approaches to healthcare, ultimately leading to more effective treatments and preventative strategies for various metabolic and immune-related conditions.

### 4.10. Strengths and Limitations

This study represents the first bibliometric and visualization analysis of macrophages associated with MetS over the past decade. We ensure the comprehensiveness and accuracy of the search methodology to include as many relevant research articles as possible. This study employed four widely recognized tools for in-depth visual analysis, not only reviewing the research progress and hot issues in the field, but also deepening our understanding of the evolution of key research nodes over time. Additionally, it provides well-founded predictions for future research trends.

However, certain limitations warrant acknowledgment. First, this study relied exclusively on data from the WoSCC database, thereby excluding relevant literature available in other databases such as PubMed, Scopus, and Cochrane Library. This reliance may have resulted in the omission of pertinent literature. Second, we only included English-language publications in our study, which might have introduced a bias in language. Last, the data set used in this study covers the period from January 1, 2014 to September 30, 2024. As 2024 is still ongoing, it is possible that additional research on macrophages associated with MetS will be published after the cutoff date, which could alter the findings of this analysis.

## 5. Conclusions

In conclusion, this study systematically evaluates the research landscape, priorities, and emerging trends of macrophages associated with MetS over the last decade. By providing a comprehensive overview of global publication patterns, this research assists scholars in identifying influential authors, institutions, and journals within this area. Furthermore, the clustering analysis of keywords and co-citations sheds light on the current research landscape and offers a strategic roadmap for exploring new research directions.

## Figures and Tables

**Table 1:** Top 10 countries/regions by publication volume and corresponding citation frequency.

Rank	Country/region	Publications	Counts (%)	Citations
1	USA (North America)	497	30.66	25,770
2	China (Asia)	387	23.87	10,558
3	Japan (Asia)	151	9.32	5702
4	Germany (Europe)	109	6.72	4785
5	Italy (Europe)	94	5.80	4316
6	South Korea (Asia)	91	5.61	2144
7	Spain (Europe)	86	5.31	3079
8	UK (Europe)	71	4.38	3148
9	France (Europe)	68	4.19	3874
10	Netherlands (Europe)	67	4.13	2619

**Table 2:** Top 11 institutions ranked by publication volume and collaborative networks.

Rank	Institution	Publications	Total link strength	Citations
1	Harvard Med Sch (USA)	30	11	867
2	Shanghai Jiao Tong Univ (China)	28	12	1345
3	Univ Sao Paulo (Brazil)	27	2	1904
4	Univ Michigan (USA)	19	10	1147
5	Chinese Acad Sci (China)	18	9	1583
6	Univ Calif San Diego (USA)	18	5	2489
7	Univ Washington (USA)	18	3	1297
8	Sichuan Univ (China)	16	3	405
9	Mayo Clin (USA)	16	2	1454
10	Univ Missouri (USA)	16	2	577
11	Radboud Univ Nijmegen (Netherlands)	16	1	1060

**Table 3:** The top 10 authors with the most publications and citations on macrophages associated with MetS.

rank	Author	Publications	Co-cited authors	Citations
1	Huang Yan	10	Gozal David	906
2	Li Yanchun	9	Joosten Leo A. B.	581
3	Gozal David	9	Blueher Matthias	562
4	Lu Zhongyang	8	Sowers James R.	439
5	Blueher Matthias	8	Tacke Frank	423
6	Lopes-Virella Maria F.	7	Eirin Alfonso	418
7	Sowers James R.	7	Lerman Amir	418
8	Montserrat-de la Paz Sergio	6	Lerman Lilach O.	418
9	Galvez Isabel	6	Tang Hui	418
10	Martin-Cordero Leticia	6	Netea Mihai G.	371

**Table 4:** The top 10 journals ranked by publication volume and evaluation indicators in 2023.

Rank	Journal	Publications	IF (JCR2023)	JCR quartile	Citations
1	Frontiers in Immunology	67	5.7	*Q*1	2501
2	International Journal of Molecular Sciences	59	4.9	*Q*1	2027
3	PLoS One	43	2.9	*Q*1	1293
4	Scientific Reports	41	3.8	*Q*1	1159
5	Nutrients	31	4.8	*Q*1	1608
6	Frontiers in Endocrinology	22	3.9	*Q*2	812
7	Food & Function	14	5.1	*Q*1	402
8	Obesity	13	4.2	*Q*1	722
9	Cell	13	45.5	*Q*1	406
10	American Journal of Physiology-Endocrinology and Metabolism	13	4.2	*Q*1	372

**Table 5:** The top 10 co-cited journals and their evaluation indicators in 2023.

Rank	Journal	Citations	IF (JCR2023)	JCR quartile
1	Journal of Clinical Investigation	2883	13.3	*Q*1
2	Diabetes	2669	6.2	*Q*1
3	Nature	2367	50.5	*Q*1
4	Journal of Biological Chemistry	2339	4.0	*Q*2
5	PLoS One	2315	2.9	*Q*1
6	Cell Metabolism	1785	27.7	*Q*1
7	Proceedings of the National Academy of Sciences of the United States of America	1782	9.4	*Q*1
8	Journal of Immunology	1699	3.6	*Q*2
9	Nature Medicine	1460	58.7	*Q*1
10	Cell	1453	45.5	*Q*1

**Table 6:** Top 15 most cited references based on co-citation analysis.

Rank	Article title	Source title	Author	Citations	Year	Document type	DOI	Main essence
1	Inflammation as a link between obesity, metabolic syndrome and type 2 diabetes	Diabetes Research and Clinical Practice	Esser et al. [18]	1331	2014	Review	10.1016/j.diabres.2014.04.006	The role of chronic low-grade inflammation in obesity, metabolic syndrome, and type 2 diabetes.
2	Vitamin C and Immune Function	Nutrients	Carr and Maggini [19]	973	2017	Review	10.3390/nu9111211	The role of vitamin C in enhancing immune function, particularly in phagocytic cells like macrophages, and its potential to improve insulin sensitivity in type 2 diabetes.
3	Liver inflammation and fibrosis	Journal of Clinical Investigation	Koyama and Brenner [20]	815	2017	Review	10.1172/JCI88881	The mechanisms, etiology, pathological processes, and potential therapeutic approaches related to liver inflammation and fibrosis.
4	Proteomic and Metabolomic Characterization of COVID-19 Patient Sera	Cell	Shen et al. [21]	799	2020	Article	10.1016/j.cell.2020.05.032	The proteomic and metabolomic changes in COVID-19 patients, revealing the impact of systemic inflammation on metabolic pathways and macrophage activation.
5	Obstructive sleep apnea syndrome	Nature Reviews Disease Primers	Lévy et al. [22]	679	2015	Article	10.1038/nrdp.2015.15	Pathophysiology, diagnosis, treatment, and impact on quality of life of obstructive sleep apnea syndrome.
6	Inflammation in obesity, diabetes, and related disorders	Immunity	Rohm et al. [23]	631	2022	Review	10.1016/j.immuni.2021.12.013	The mechanisms underlying inflammation in obesity, T2D and related disorders.
7	Obesity and Cancer Mechanisms: Tumor Microenvironment and Inflammation	Journal of Clinical Oncology	Iyengar et al. [24]	569	2016	Review	10.1200/JCO.2016.67.4283	The mechanisms linking obesity to cancer, focusing on the role of inflammation and macrophage polarization in the tumor microenvironment.
8	Metabolic Dysfunction Drives a Mechanistically Distinct Proinflammatory Phenotype in Adipose Tissue Macrophages	Cell Metabolism	Kratz et al. [25]	567	2014	Article	10.1016/j.cmet.2014.08.010	Adipose tissue macrophages in obese individuals exhibit a unique “metabolically activated” proinflammatory phenotype.
9	Targeting inflammation in the treatment of type 2 diabetes: time to start	Nature Reviews Drug Discovery	Donath [26]	543	2014	Review	10.1038/nrd4275	The potential of anti-inflammatory therapies for type 2 diabetes.
10	Modulation of gut microbiota during probiotic-mediated attenuation of metabolic syndrome in high fat diet-fed mice	Isme Journal	Wang et al. [27]	520	2015	Article	10.1038/ismej.2014.99	The modulatory effects of three probiotic strains on the gut microbiota of mice with metabolic syndrome induced by a high-fat diet.
11	Gut microbiota-bile acid-interleukin-22 axis orchestrates polycystic ovary syndrome	Nature Medicine	Qi et al. [28]	409	2019	Article	10.1038/s41591–019–0509–0	The gut microbiota–bile acid–interleukin-22 axis plays a key regulatory role in the development and progression of polycystic ovary syndrome.
12	The Macrophage Switch in Obesity Development	Frontiers In Immunology	Castoldi et al. [29]	392	2016	Review	10.3389/fimmu.2015.00637	Macrophages are central mediators of obesity-induced AT inflammation and insulin resistance.
13	Genetics and biology of pancreatic ductal adenocarcinoma	Genes & Development	Ying et al. [30]	378	2016	Review	10.1101/gad.275776.115	The complex genetic mutations, metabolic alterations, and immune regulatory mechanisms within the tumor microenvironment of pancreatic ductal adenocarcinoma.
14	Interleukin-18: Biological properties and role in disease pathogenesis	Immunological Reviews	Kaplanski [31]	374	2018	Review	10.1111/imr.12616	The biological properties of Interleukin-18 and its role in the pathogenesis of various diseases, including infectious diseases, metabolic diseases, and inflammatory diseases.
15	OLT1177 a ß-sulfonyl nitrile compound, safe in humans, inhibits the NLRP3 inflammasome and reverses the metabolic cost of inflammation	Proceedings of the National Academy of Sciences of the United States of America	Marchetti et al. [32]	366	2018	Article	10.1073/pnas.1716095115	A *β*-sulfonyl nitrile compound named OLT1177 can inhibit the activation of the NLRP3 inflammasome and reverse the metabolic burden caused by inflammation.

**Table 7:** Details of keywords with a frequency of ≥100 occurrences.

Rank	Keyword	Occurrences	Total link strength
1	Metabolic syndrome	772	5081
2	Inflammation	634	4358
3	Insulin-resistance	551	3928
4	Obesity	533	3920
5	Macrophage	483	3278
6	Expression	269	1817
7	Adipose-tissue	255	1792
8	Activation	175	1157
9	Oxidative stress	167	1096
10	Atherosclerosis	163	1093
11	Cells	125	812

## Data Availability

The data that support the findings of this study are available from the corresponding author upon reasonable request.
